# Genomics applied to the treatment of breast cancer

**DOI:** 10.18632/oncotarget.27102

**Published:** 2019-07-30

**Authors:** Diaddin Hamdan, Thi Thuy Nguyen, Christophe Leboeuf, Solveig Meles, Anne Janin, Guilhem Bousquet

**Affiliations:** ^1^ Hôpital La Porte Verte, Versailles F-78004, France; ^2^ U942, Université Paris-Diderot, INSERM, Paris F-75010, France; ^3^ National Cancer Hospital, Medical Oncology Department 2, Ha Noi 110000, Viet Nam; ^4^ Ha Noi Medical University, Oncology Department, Ha Noi 116001, Viet Nam; ^5^ AP-HP-Hôpital Saint-Louis, Laboratoire de Pathologie, Paris F-75010, France; ^6^ AP-HP-Hôpital Avicenne, Service d’Oncologie Médicale, Bobigny F-93000, France; ^7^ Université Paris 13, Leonard de Vinci, Villetaneuse F-93430, France

**Keywords:** constitutional genomics, genomics applied to treatment, genomics of breast cancer, molecular and histological classification, tumor heterogeneity

## Abstract

Breast cancer remains a major health issue in the world with 1.7 million new cases in 2012 worldwide. It is the second cause of death from cancer in western countries. Genomics have started to modify the treatment of breast cancer, and the developments should become more and more significant, especially in the present era of treatment personalization and with the implementation of new technologies. With molecular signatures, genomics enabled a de-escalation of chemotherapy and personalized treatments of localized forms of estrogen-dependent breast cancers. Genomics can also make a real contribution to constitutional genetics, so as to identify mutations in a panel of candidate genes. In this review, we will discuss the contributions of genomics applied to the treatment of breast cancer, whether already validated contributions or possible future applications linked to research data.

## INTRODUCTION

Breast cancer is the first cancer in terms of incidence among women, with 1.7 million new cases in 2012 worldwide. It is also the second cause of death from cancer in western countries with 40,000 deaths per year [1, 2]. In the last 20 years, breast cancer mortality has continuously decreased as a result of mass screening programs and early diagnosis, but also as a consequence of improved treatment for both localized and metastatic disease [3, 4].

In the era of personalized cancer medicine, advances in genomics are essential assets. In this review, we will address current knowledge in genomics applied to the treatment of breast cancers.

## HISTOLOGIC AND MOLECULAR CLASSIFICATIONS

**Figure 1:**
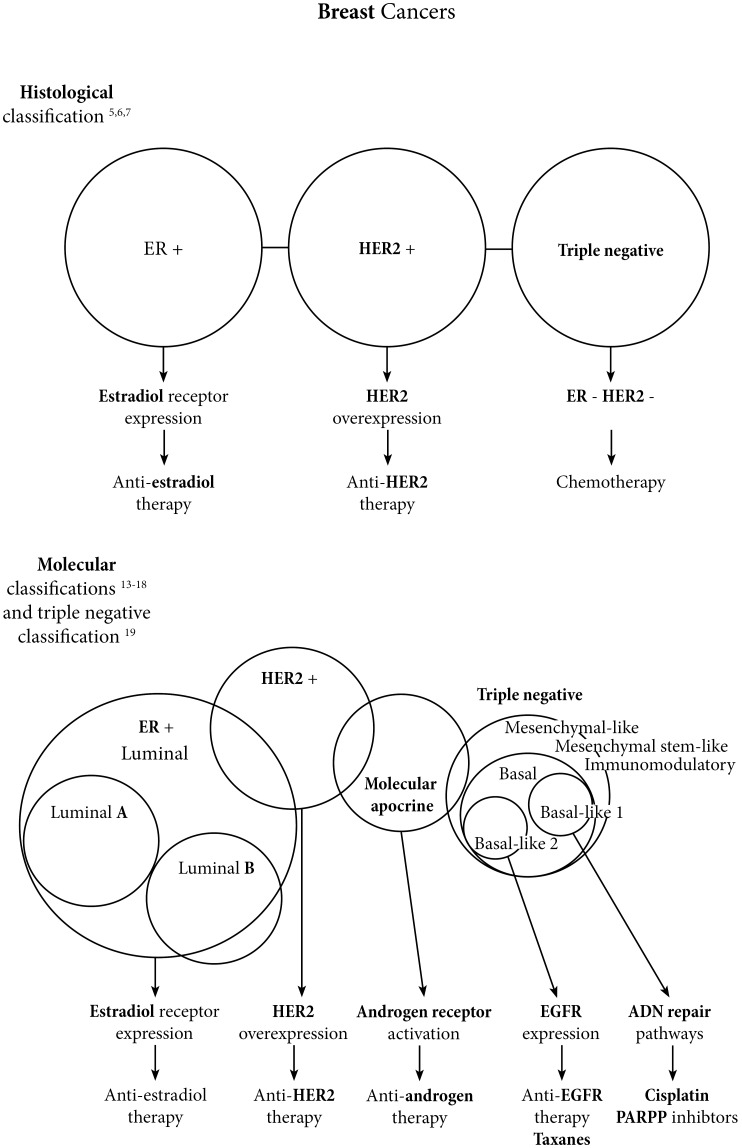
Breast cancers landscape evolution from histologic to molecular classifications.

There are schematically three main histologic types of breast cancer (Figure 1): i) estrogen-dependent breast cancers expressing the estradiol receptor (ER) and treated with a panel of drugs that target the estradiol receptor pathway [5]; ii) breast cancers overexpressing the human epidermal growth factor receptor 2 (HER2) oncoprotein and treated with anti-HER2-based chemotherapies, the first anti-HER2 being a therapeutic monoclonal antibody, trastuzumab [6]; and iii) “triple negative” breast cancers which lack the expression of the estradiol receptor, the progesterone receptor, and HER2. There are still no targeted therapies for triple-negative breast cancers, which have a high metastatic potential, and consequently a bad prognosis [7].

Since 2000, several molecular subtypes have been identified, including the aforementioned histological subtypes (Figure 1). Peru *et al.* identified six molecular sub-types: Luminal A, Luminal B, Luminal C, HER2-enriched, Basal-like and Normal-like [8–10]. The luminal A subtype has the best prognosis and the lowest proliferative potential, including highly estrogen-dependent breast cancers. Luminal B subtype is also estrogen-dependent, but to a lesser extent, and corresponds to a sub-group of more aggressive cancers with a higher proliferative potential [11]. For the basal-like subtype, 50 to 75% are triple-negative breast cancers. Their aggressiveness is frequently the result of the loss of the retinoblastoma protein 1 (pRb1), of a high rate of tumor protein 53 (*TP53*) gene mutations and also of mutations in genes involved in DNA-repair mechanisms, such as breast cancer type 1 susceptibility (*BRCA1*) gene [12].

This early molecular classification was later revised, with the following subtypes: Luminal A and B, HER2-positive/ER-positive, HER2-positive/ER-negative, basal p53-altered and basal p53-normal (Figure 1). In this more recent version, 7% of cancers are of unknown sub-type because of the heterogenic expression of markers [13, 14]. Since 2005, among ER-negative cancers, several teams have identified a “molecular apocrine” subtype, characterized by an activation of the androgen receptor (AR) pathway and by the expression of AR target genes in 50% of cases, or by HER2 overexpression [15–17]. In 2012, a new molecular classification was established which includes Luminal, molecular Apocrine and Basal-like subtypes (Figure 1) [18]. Different immunohistochemistry markers have been proposed for *in situ* characterization of molecular apocrine breast cancers, including androgen receptor (AR) and gross cystic disease fluid protein 15 (GCDFP15) [17].

In 2011, Lehman *et al*. proposed a molecular classification of triple-negative breast cancers which we will discuss later [19].

## MOLECULAR SIGNATURES AND ESTROGEN-DEPENDENT BREAST CANCERS

For estrogen-dependent breast cancers, the contribution of genomics has been small. However, molecular signatures have substantially modified clinical practice, enabling the therapeutic decision for adjuvant chemotherapy to be redefined for localized breast cancers [20]. Three commercially available tests are currently used: MammaPrint^®^ (Agilent, The Netherlands) [21, 22], OncotypeDx^®^ [23] and PAM50 (Prediction of Microarray using 50 classifier genes plus 5 reference genes) (Prosigna^®^ kit) (see Table 1) [24, 25]. These tests quantify the expression levels of a limited panel of genes in the primary tumor. Most of them were developed for formalin-fixed paraffin-embedded tissue samples for implementation in daily practice.

**Table 1:** Gene panel tests used for therapeutic decision of localized breast cancers (Adapted from [28])

Signature	Number of genes	Clinical application	Risk category	References
MammaPrint	70	N−, ER+ or ER− Estimates relapse risk	Low and high	[22]
OncotypeDX	21	ER+, HER2−, N− Estimates chemotherapy benefit and relapse risk during hormonotherapy	Low, intermediate and high	[23]
EndoPredict	11	ER+, HER2−, N− or N+ Predicts local and metastatic relapse during hormonotherapy	Low and high	[74]
Prosigna (PAM50)	50	ER+/N− and N+ treated by hormonotherapy Predicts 10-year metastasis-free survival	Low, intermediate and high	[25]
Breast Cancer Index	5 and 2 genes ratio	ER+, N− Estimates metastatic risk Predicts late metastatic risk and efficacy of prolonged hormonotherapy	Low and high	[75]
Rotterdam	76	ER+, N− Predicts relapse under treatment with tamoxifen	Low and high	[76]
BluePrint	80	Discriminates sub-types with different level of sensitivity to adjuvant treatment	Not applicable	[77]

N: Node status in TNM classification; ER: Estradiol Receptor; RT-PCR: Reverse Transcription-Polymerase Chain Reaction.

These molecular signatures are used to classify patients according to their risk of metastatic relapse, to guide the decision for adjuvant chemotherapy when conventional criteria are insufficient [20]. This is particularly true for ER-positive, HER2-negative breast cancers without lymph node involvement (N0): in this sub-group of patients, adjuvant chemotherapy significantly reduces the risk of metastatic relapse only for high-risk patients [26–28]. The OncotypeDx^®^ signature comprises three risk categories, raising the question of how to treat «intermediate-risk» patients. Recently, the TAILORx study clearly demonstrated the absence of benefit from adjuvant chemotherapy in this subgroup of intermediate-risk patients [29]. A meta-analysis of 147 articles concluded that molecular signatures for breast cancer enable 10% of patients at high clinical risk of relapse to be reclassified as low-risk patients, thus reducing the use of chemotherapy, with a favorable cost-efficiency ratio and improved quality of life for non-treated patients [30].

For estrogen-dependent breast cancers, the other contributions of genomics remain in the research field. For example, recent studies have identified the presence of mutations of the estrogen receptor 1 (*ESR1*) gene, such as the D538G or Y537S/C/N mutations, associated with resistance to anti-estrogens. These mutations change the conformation of the ligand binding site, thereby reducing the affinity of tamoxifen for the estrogen receptor [31, 32]. The systematic screening for these mutations is not currently recommended. Further studies are required to demonstrate their possible usefulness in guiding hormone-therapy prescription in daily practice.

## GENOMICS APPLIED TO THE TREATMENT OF HER2-OVEREXPRESSING BREAST CANCERS

It is recommended to determine HER2 status in the primary tumor or in metastatic samples using a standardized immunostaining method. For doubtful cases, *in situ* hybridization methods are currently used to determine the *HER2* gene copy number [33, 34]. In 2013, an international consensus clearly defined the criteria for HER2 protein overexpression and for *HER2* gene amplification [35].

However, these methods entail certain limitations, typically for micro-invasive foci in a primary tumor or a micro-metastatic axillary lymph node [36].

Digital droplet PCR (ddPCR), easier to implement than *in situ* hybridization methods, seems to be a reliable alternative for the evaluation of the *HER2* copy number in breast or gastric cancers [37–39]. A major limitation could be intra-tumor heterogeneity. Indeed, primary breast cancers are heterogeneous [28], and this is also the case for HER2 status [40]. Combining laser-microdissection with ddPCR overcomes this limitation, by enabling a precise assessment of the *HER2* copy number within a cancer sample. In a recent study, we validated the use of laser microdissection combined with ddPCR to assess *HER2* copy number in micro-invasive breast cancers with at least 50 invasive cancer cells. We then applied this methodology to a 45-year-old patient with extensive *in situ* breast cancer, and no associated micro-invasion except a micro-metastasis found only on one section of the sentinel axillary lymph node. We first laser-microdissected the micro-metastatic foci, and then used ddPCR to demonstrate that *HER2* was amplified. This led us to optimize the adjuvant treatment for our patient, and she received trastuzumab-based adjuvant chemotherapy [37].

**Figure 2:**
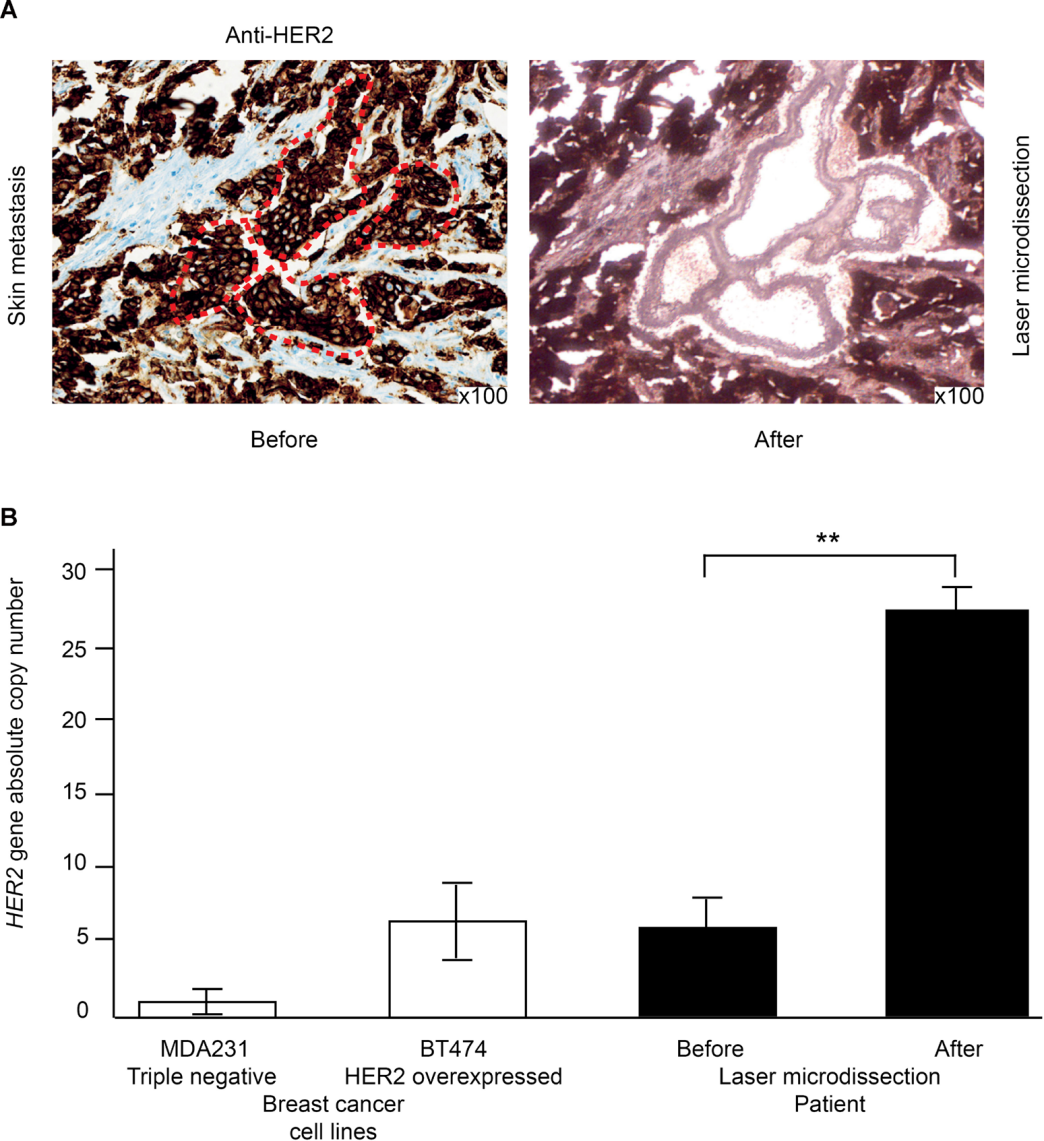
Laser-microdissection of cancer cells combined with ddPCR to precisely assess HER2 amplification level on a skin metastasis of recurrent HER2-overexpressing breast cancer. (**A**) HER2 is typically overexpressed using immunohistochemistry (left panel). The right panel shows the laser-microdissected HER2-overexpressing cancer cells. (**B**) the *HER2* copy number is much higher in the laser-microdissected cells than in the whole tumor. MDA231 triple-negative breast cancer cell lines serve as a negative control while the BT474 HER2-overexpressing cancer cell line serves as a positive control.

For HER2-overexpressing breast cancers, another issue remains to be deciphered: is there any clinical benefit to be drawn from the systematic determination of *HER2* amplification level within the tumor? Singer *et al.* demonstrated a correlation between the level of *HER2* amplification and the response to anti-HER2 treatments in the neoadjuvant setting [41]. Laser-microdissection of cancer cells from a metastatic tumor biopsy combined with ddPCR could be used to overcome signal dilution by enrichment with tumor cells. So, in a skin metastasis of a HER2-expressing breast cancer, we have demonstrated that *HER2* copy number evaluated by ddPCR passed from 6 to 34, without and with prior laser microdissection respectively (Figure 2).

## GENOMICS APPLIED TO THE TREATMENT OF TRIPLE-NEGATIVE BREAST CANCERS

Triple-negative breast cancers remain a therapeutic challenge. Lehmann *et al.* established a molecular classification with six molecular subtypes to personalize the treatment of these breast cancer sub-types: Basal-like 1 (BL1), Basal-like 2 (BL2), Immunomodulatory (IM), Mesenchymal-like (M), Mesenchymal stem-like (MSL), and Luminal androgen receptor (LAR) (Figure 1) [19]. In the neoadjuvant setting, Masuda *et al*. demonstrated significant associations between these molecular subtypes and pathological complete response under chemotherapy. BL1 subtype is associated with greater chemo-sensitivity, whereas BL2 and LAR subtypes are more chemo-resistant subtypes [42].

Unfortunately, this classification still has few clinical applications, even in clinical trials dedicated to triple-negative breast cancers. For example, the BL2 subtype, characterized by an activation of the epidermal growth factor pathway, could respond to anti-epidermal growth factor receptor (EGFR) therapies. A phase II study assessed the benefit of cetuximab, a monoclonal anti-EGFR antibody, in the treatment of 173 women with metastatic triple-negative breast cancers. The results were disappointing, with a non-significant survival gain of 2.2 months [43]. For anti-EGFR-based treatments, a study could be dedicated exclusively to BL2 subtypes, ideally with high *EGFR* copy numbers and a quadruple wild-type status of *KRAS*, *NRAS*, *BRAF,* and *PIK3CA* genes, as for colon cancers [43, 44].

The same arguments can be applied to the LAR subtype, characterized by androgen receptor signaling pathway activation, and corresponding to certain molecular apocrine sub-types [16]. The LAR subtype accounts for 11% of triple-negative breast cancers [19]. In two phase II studies using anti-androgens for the treatment of patients with metastatic triple-negative breast cancers, disease stabilization at 6 months was observed for less than 20% of patients [45, 46]. It should be noted that the patients were not pre-selected according to LAR subtype.

In 2012 and 2013, we conducted a pilot study among five women with metastatic triple-negative breast cancers. For the five patients, we performed transcriptomic analyses on metastasis biopsies, and classified their respective metastatic cancers according to Lehmann’s classification. We also established individual xenograft models from the same metastasis biopsies. For each patient xenograft model, we tested a panel of drugs or drug combinations, guided by transcriptomic data. One patient was classified BL2, with EGF pathway activation and no mutation of the EGF pathway genes. In the corresponding xenograft, the most effective regimen was a combination of paclitaxel and cetuximab. This regimen was offered to the patient as a third-line resort treatment with almost complete metabolic response [47]. On the basis of transcriptomic analyses and chemosensitivity data obtained from the different xenografts, we personalized the resort treatment for the four other women in our study. In all cases, despite the fact that this resort treatment was third-line or fourth-line, the time-to-progression was longer than that observed with previous lines of treatment [48].

## GENOMICS AND TUMOR HETEROGENEITY

Tumor heterogeneity is probably insufficiently taken into account in daily clinical practice, particularly for the treatment of metastatic disease. Most molecular analyses are performed on primary tumors, even in metastatic stages. However, metastatic clones can be a minority in the primary tumors they are deriving from [49, 50]. For HER2 status assessment, we have previously noted the benefit of combining molecular and tissue analyses, particularly with the contribution of laser-microdissection to overcome the limitation of tumor heterogeneity [37–39, 51, 52].

Molecular analyses on metastases are rare, mainly because of difficulties in obtaining these samples, and despite the fact that radiology-guided biopsies have considerably reduced this limitation [53]. For breast cancer, eleven studies have been dedicated to whole-genome analyses of metastatic biopsies (Table 2). In addition, most of these studies only included small numbers of patients, and the genome analyses were generally performed on tumors that were not laser-microdissected. One large study included 216 metastatic samples, some of them paired with samples from the corresponding primary breast cancers. They showed that metastatic clones are enriched with certain molecular abnormalities compared to the primary tumors [54].

**Table 2:** Genomics studies on breast cancer metastasis samples

Number of samples	References
8	Weigelt, *Proc Natl Acad Sci U S A*. 2003 [78]
14	Wang, *Genes Chromosomes Cancer* 2009 [79]
30	Desouki, *J Cancer Res Clin Oncol* 2011 [80]
14	Craig, *Mol Cancer Ther* 2013 [81]
15	Lee, *Oncotarget* 2015 [82]
62	Onstenk, *Cancer Lett* 2015 [83]
13	McBryan, *Clin Cancer Res* 2015 [84]
55	Lang, *Breast Cancer Res Treat* 2015 [85]
80	Kimbung, *Clin Cancer Res* 2016 [86]
88	Fumagalli, *Ann Oncol* 2016 [87]
216	Lefebvre, *PLoS Med* 2016 [54]

Our research team is conducting a program on brain metastases. As part of this program, we performed transcriptomic analyses on laser-microdissected metastatic lymph-nodes of 28 women with HER2-overexpressing or triple negative metastatic breast cancers. Supervised analyses compared the transcriptomic profiles of women who developed brain metastases with those who did not. We identified *CDKN2A*/p16 as a gene associated with the risk of brain metastases and decreased survival [55].

## GENOMICS AND CONSTITUTIONAL GENETICS OF BREAST CANCER

In the last ten years, a panel of genes has been proposed for the diagnosis of hereditary familial cancers. In the context of hereditary predisposition for breast and/or ovarian cancers, the United States National Comprehensive Cancer Network recommends a panel of nineteen genes and proposes corresponding clinical screening tests (Table 3) [56]. In France, the Genetics and Cancer Group, supported by the French National Cancer Institute, recently updated their recommendation to test a panel of 13 genes accompanied by prevention and screening measures for patients and their families (Tables 4 and 5) [57, 58]. With twenty-eight platforms covering the French territory and dedicated to molecular biology, patients at risk for hereditary cancer can have the benefit of recent technologies applied to constitutional genetics. These platforms have implemented high-throughput sequencing tools like the Next Generation Sequencing (NGS) systems, to identify mutational hot-spots in a panel of high penetrance genes. Several types of NGS sequencers are currently used for routine care and also for research purposes, such as Illumina, Applied Biosystems SOLiD System, 454 Life Sciences (Roche), Helicos HeliScope, Complete Genomics, Pacific Biosciences PacBio and Life Technologies Ion Torrent [59].

**Table 3:** National Comprehensive Cancer Network guidelines for breast and ovarian cancer management based on genetic and familial high-risk assessment (Adapted from [56])

Gene	Breast cancer risk management	Ovarian cancer risk management	Other cancer risk management
*ATM*	Increased risk Annual mammography and breast MRI starting at **40 years** RRS: based on FH	Potential increase in risk with insufficient evidence to recommend RRS	Insufficient evidence for pancreas or prostate cancers
*BRCA1*	Increased risk **25–29 years**, annual breast MRI or mammogram **30–75 years**, annual mammogram and breast MRI **>75 years**, based on IR RRS: based on IR and FH	Increased risk RRS: based on individual risk and FH between **35-40 years**	Prostate, uterine (possible)
*BRCA2*	Increased risk **25–29 years**, annual breast MRI or mammogram **30–75 years**, annual mammogram and breast MRI **>75 years**, based on IR RRS: based on IR and FH	Increased risk RRS: based on IR and FH between **40-45 years**	Pancreas, prostate, melanoma
*PALB2*	Increased risk Annual mammography and MRI starting at age **30 years** RRS: based on FH	Insufficient evidence	Insufficient evidence
*TP53*	Increased risk **20–29 years**, annual breast MRI **30–75 years**, annual breast MRI and mammogram **>75 years**, based on IR RRS: based on IR and FH	No increased risk	Neurological cancers, colon, skin cancers
*CDH1*	Increased risk for lobular cancer Annual mammogram and breast MRI starting at age **30 years** RRS: based on FH	No increased risk	Diffuse gastric cancer
*PTEN*	Increased risk Annual mammogram with breast MRI starting at age **30–35 years** or 5-10 years before earliest breast cancer in family **>75 years**, based on IR RRS: based on IR and FH	No increased risk	Endometrial cancer, thyroid, colon, renal cancer, skin cancers
*BRIP1*	Insufficient evidence	Increased risk Consider RRS at **45–50 years**	Not available
*CHEK2*	Increased risk Annual mammogram and breast MRI starting at age **40 years** RRS: based on FH	No increased risk	Colon cancer
*NBN*	Increased risk Annual mammogram and breast MRI starting at age **40 years** RRS: based on FH	Insufficient evidence	Insufficient evidence
*NF1*	Increased risk Annual mammogram from age **40 years** and consider breast MRI from **30–50 years** RRS: based on FH	No increased risk	Malignant peripheral nerve sheath tumors, GIST, others
*STK11*	Increased risk Annual mammogram and breast MRI starting at age **25 years** RRS: based on FH	Increased risk of non-epithelial cancers Annual pelvic examination and PAP smear	Colon, stomach, pancreas, cervix, uterine, testis, lung
*RAD51C*	Insufficient evidence	Increased risk Consider RRS at **45–50 years**	Not available
*RAD51D*	Insufficient evidence	Increased risk Consider RRS at **45–50 years**	Not available
*MLH1*	Insufficient evidence	Increased risk	Colon, uterine, others
*MSH2*	manage based on FH		
*MSH6*			
*PMS2*			
*EPCAM*			

MRI: Magnetic Resonance Imaging; RRS: Risk reduction surgery; FH: Family History; IR: Individual Risk.

**Table 4:** Recommendations of the Cancer and Genetics Group and the French National Institute of Cancer concerning gene panel analyses in the context of a hereditary predisposition to breast and ovarian cancers (Adapted from [57])

Gene	Cytogenetic location	Penetrance	Protein functions	Cumulate risk of breast cancer	References
*BRCA1*	17q21.31	High	Repair of DNA double-strand breaks using homologous recombination, cell cycle control, maintaining of genome integrity	46–87% lifetime risk	[88, 89]
*BRCA2*	13q13.1	High	Repair of DNA double-strand breaks using homologous recombination	38–84% lifetime risk	[88, 90]
*PALB2*	16p12.2	Moderate	Partner of *BRCA2* and regulator of its stability and its nuclear localization	35% at 70 years	[91, 92]
*TP53*	17p13.1	High	Transcription Factor, cellular cycle, apoptosis, senescence, DNA Repair	80% life-time risk (premenopausal)	[93, 94]
*CDH1*	16q22.1	Moderate	E-cadherin, cellular adhesion molecule	39–52% before 40 years (lobular cancer)	[95, 96]
*PTEN*	10q23.31	High	Tumor phosphatase suppressor inhibiting PI3K and MAPK pathways	25–50% lifetime risk	[97, 98]
*RAD51C*	17q22	Moderate	Repair of DNA using homologous recombination in interaction with *BRCA1/2*	Not known	[99–102]
*RAD51D*	17q12	Moderate	Repair of DNA using homologous recombination and maintaining of telomere	Not known	[103–105]
*MLH1*	3p22.2	High	Mismatch repair system	5–18%	[106–111]
*MSH2*	2p21-p16				
*MSH6*	2p16.3	Moderate		Not known	
*PMS2*	7p22.1				
*EPCAM*	2p21		Partner of *MSH2*		

**Table 5:** Screening or prevention recommendations for persons carrying mutations of genes analyzed in the Cancer and Genetics Group panel (Adapted from [58])

Gene	Breast surveillance	Risk reduction surgery		Gynecologic surveillance
		Breast	Pelvis	
*BRCA1* *BRCA2*	**30 - 65 year**, annual MRI + mammogram (± Ultrasound) After **65 years**, mammogram ± Ultrasound (Recommendations HAS 2014^*^ and INCa 2017^**^)	Prophylactic mastectomy (Recommendations HAS 2014 ^*^ and INCa 2017^**^)	Prophylactic annexectomy (discussed from the age of **40 years** and according to mutations and FH of OC) ^**^	Before RRS: standard surveillance and no efficacious ovarian screening available ^*^
*PALB2* *CDH1*			No specific gynecological guidelines If FH of OC: MDC	Standard surveillance
*PTEN*				Standard surveillance If gynecologic lesions of CD: MDC
*TP53*	Starting at **20 years,** annual MRI + Ultrasound (no systematic mammogram)			Standard surveillance
*RAD51C RAD51D*	No specific breast surveillance To be adapted to FH of BC according to guidelines HAS 2014^*^	Not indicated	Prophylactic annexectomy (discussed from the age of **45 years** and according to FH of OC)	Before RRS: standard surveillance and no efficacious ovarian screening to be proposed ^**^
*MLH1* *MSH2* *MSH6* *PMS2* *EPCAM*			Ovarian and/or uterine RRS To be discussed in MDC according to Lynch syndrome guidelines	Uterine surveillance according to Lynch syndrome guidelines

BC: Breast Cancers; OC: Ovarian Cancers; FH: Family History; CD: Cowden disease; MDC: Multidisciplinary Committee; RRS: Risk reduction surgery.

^*^Recommendation of French Health Authority (HAS) 2014: French Breast cancer National screening program.

^**^©/Recommendation of French National Cancer Institute (INCa), April 2017.

In sporadic breast cancers, NGS sequencing enables four subtypes to be discriminated on the basis of different genetic and epigenetic modifications, with three genes (*PIK3CA*, *TP53,* and *GATA3*) that are modified in more than 10% of patients. Basal-like breast cancers typically harbor mutations in the *TP53, RB1,* and *BRCA1* genes, together with *MYC* amplifications [60].

Because of the potential therapeutic applications, the identification of inactivating mutations in the *BRCA1* gene, a tumor suppressor gene, is important. *BRCA1,* because of its critical role in DNA repair mechanisms through homologous recombination, is one of the most important genes associated with hereditary breast cancer [61]. More than 75% of *BRCA1*-mutated breast cancers have a triple-negative phenotype and are classified as basal-like [8]. Constitutional *BRCA1* mutations are of high penetrance, occur in 10% of breast cancer patients and in 20% of young women with triple-negative breast cancers [62]. *BRCA1* sporadic mutations are also found in 1% of breast cancers, and the promoter can be hypermethylated in 11 to 14% of cases, resulting in *BRCA1* gene inactivation [63–65].

A deficit in homologous recombination *via* *BRCA1* inactivation has provided the rationale to concomitantly inhibit other DNA repair pathways, particularly the (ADP-ribose) polymerase (PARP) enzyme pathway. Olaparib, a PARP inhibitor, showed considerable benefit in patients with metastatic *BRCA1*-mutated breast cancers [66, 67]. We, therefore, need to look for *BRCA1* germline mutations in patients at risk for hereditary breast cancer. For sporadic breast cancer, particularly for the triple negative sub-type, we also need to identify *BRCA1*-inactivating mutations in tumors.

In this domain of constitutional genetics, many questions remain unanswered, particularly the translational value of identifying mutations of unknown significance in genes of low to moderate penetrance. The contributions of Genome-Wide Association Studies (GWAS) have not been very great. To date, more than sixty GWAS have been conducted on breast cancer samples. A meta-analysis of these GWAS identified 84 loci of interest possibly associated with an increased risk of breast cancer [68, 69]. Numerous low penetrance variants have been identified, without validating their functional significance. One of these variants concerns the oncogene *FGFR2*, the FGFR2 protein being overexpressed in 5% of breast cancers. This variant corresponds to a single nucleotide polymorphism (SNP) that affects the binding site of FGFR2, thus activating the downstream signaling pathway in a ligand-independent manner [70]. It is necessary to address the potential benefit of targeting FGFR2 for therapeutic purpose. Another SNP, in the 8q24 region, participates in regulating *MYC* oncogene transcription which is distant from this SNP by more than 300 kb [71]. Most GWAS studies suggest that mutations in low penetrance genes could partially explain genetic predisposition to breast cancer, even though their functional significance remains unclear [72].

## CONCLUSIONS

The contribution of genomics applied to the treatment of breast cancer remains moderate. In practice, it is limited to informing adjuvant treatment decisions for early-stage diseases and to HER2-overexpressing breast cancers whatever the stage. However, breast cancers are heterogeneous and complex. Treatments need to be adjusted according to molecular subtypes and guided by the underlying genetic events. Several programs are ongoing to map the complex genetics of breast cancer, using multi-omic approaches such as the Molecular Taxonomy of Breast Cancer International Consortium (METABRIC) [73], which will help to take tumor heterogeneity into account more efficiently. Also, the increasingly widespread utilization of NGS will help to decipher the individual molecular complexity of breast cancers. This will rapidly increase the contribution of genomics in shaping breast cancer treatment in the next few years, especially in the present era of personalized treatments.

## Abbreviations

ER: estradiol receptor
HER2: human epidermal growth factor receptor 2
Rb1: retinoblastoma protein 1; *TP53*: tumor protein 53 gene; *BRCA1*: breast cancer type 1 susceptibility gene
AR: androgen receptor
GCDFP15: gross cystic disease fluid protein 15
PAM50: Prediction of Microarray using 50 classifier genes plus 5 reference genes
*ESR1*: estrogen receptor 1 gene
ddPCR: digital droplet polymerase chain reaction
BL1: basal-like 1
BL2: basal-like 2
IM: immunomodulatory
M: mesenchymal-like
MSL: mesenchymal stem-like
LAR: luminal androgen receptor
EGFR: epidermal growth factor receptor
NGS: next generation sequencing
PARP: ADP-ribose polymerase
GWAS: genome-wide association studies
SNP: single nucleotide polymorphism
METABRIC: Molecular Taxonomy of Breast Cancer International Consortium
N: nodes
TNM: tumor, node, metastases
RT-PCR: Reverse Transcription-Polymerase Chain Reaction
MRI: Magnetic Resonance Imaging
RRS: Risk reduction surgery
FH: Family History
IR: Individual Risk
BC: Breast Cancers
OC: Ovarian Cancers
CD: Cowden disease
MDC: Multidisciplinary Committee
HAS: French Health Authority
INCa: French National Cancer Institute
